# The efficacy of single posterior debridement, bone grafting and instrumentation for the treatment of thoracic spinal tuberculosis

**DOI:** 10.1038/s41598-021-83178-0

**Published:** 2021-02-11

**Authors:** Zhi Yi, Qichun Song, Jiao Zhou, Yongchun Zhou

**Affiliations:** 1 Department of Orthopedic, Shaanxi Provincial People’s Hospital, 256# You-yi West Road, Xi’an, 710068 Shaanxi People’s Republic of China; 2 Department of Orthopedic, 2nd Affiliated Hospital of Xi’an Jiaotong University, Xi’an, Shaanxi People’s Republic of China; 3 Department of Surgery Center, Shaanxi Provincial People’s Hospital, Xi’an, Shaanxi People’s Republic of China

**Keywords:** Tuberculosis, Therapeutics

## Abstract

The aim of this study was to investigate the clinical efficacy of single posterior debridement, bone grafting and instrumentation for the treatment of thoracic spinal tuberculosis in adult patients. A retrospective analysis was conducted between June 2013 and September 2017of 88 adult patients with thoracic spinal tuberculosis. All patients were treated with single posterior debridement, bone grafting and instrumentation. The clinical manifestations and laboratory and imageological results were subsequently analysed. All patients were followed for 40.6 ± 4.1 months (range, 36–48 m). Bony fusion was achieved in all bone grafts of thoracic vertebrae. The visual analogue scale scores, erythrocyte sedimentation rate and C-reactive protein levels 6 weeks after surgery and at the final follow up were significantly lower than the preoperative levels (*P* < 0.05). The postoperative and final follow up kyphosis angles were both significantly smaller than the preoperative kyphosis angles (*P* < 0.05). The postoperative angle correction rate reached 81.5% and the postoperative angle loss reached only 4.1%. At the last follow up, American Spinal Injury Association improvement was significant, compared with the preoperative levels (*P* < 0.05). The single posterior approach can achieve satisfactory clinical outcomes in the treatment of thoracic spinal tuberculosis.

## Introduction

The incidence of spinal tuberculosis (TB) is increasing in developing countries and 2 to 3 million deaths worldwide are related to spinal TB every year^1–3^. Spinal TB is the most common skeletal tuberculosis, accounting for approximately 50% of all skeletal tuberculosis cases^4^. Spinal infections can cause destruction and collapse of the vertebral body and lead to kyphosis and neurological impairment^5^. When the blood supply to the thoracic spinal cord is inadequate because of thoracic spinal canal stenosis, patients experiencing severe destruction of bone or spinal instability are more prone to neurological impairment^6, 7^.

Although chemotherapy plays a crucial role in spinal TB treatment, it is often necessary to correct kyphosis and improve neurological function through surgical intervention. Debridement, kyphosis correction and reconstruction of the spine are widely used as methods for surgical treatment of spinal tuberculosis^8^. The optimal surgical treatment for spinal TB remains controversial. The single anterior approach allows for direct debridement, bone grafting and instrumentation, yet the outcomes of this approach regarding kyphosis correction and maintenance are not satisfactory^8, 9^. The single posterior approach is a proven spinal TB treatment with favourable outcomes^10^; however, it still does not achieve thorough debridement of lesions in the anterior spine. Some surgeons have reported the performance of this one-stage surgery via the posterior approach alone^10, 11^. However, there are still few known studies with a long-term follow up in thoracic spinal TB. This study was designed to analyse the clinical outcomes of single posterior debridement, bone grafting and instrumentation in the treatment of adult patients with thoracic spinal TB with a maximum 3-year follow up in 88 patients.

## Patients and methods

### Basic information

This retrospective study included 88 active pulmonary TB-free patients who were diagnosed with active spinal TB between June 2013 and September 2017. Clinical diagnosis of active spinal TB was based on clinical symptoms, laboratory findings (erythrocyte sedimentation rate [ESR] and C-reactive protein [CRP] level); radiographic examination (x-ray, computed tomography (CT) and magnetic resonance imaging [MRI]) and pathological examination. Other inclusion criteria were (1) monosegmental thoracic spinal TB, with the destruction limited to a single vertebral segment and affecting no more than one vertebral motor unit; (2) progressive neurological impairment; (3) poor outcomes of conservative treatment and (4) serious kyphosis or progression of kyphosis. Exclusion criteria were (1) postoperative recurrence of thoracic spinal TB; (2) thoracic spinal TB combined with serious osteoporosis; (3) thoracic spinal TB combined with cancer or degeneration of the intervertebral disc that could affect the evaluation of clinical outcomes and (4) thoracic spinal TB combined with severe tuberculosis in other organs (e.g. pulmonary/renal tuberculosis) in a poor nutritional state that might affect the evaluation of clinical outcomes. Ethical approval from the Ethics Committee of the Shaanxi Provincial People’s Hospital was obtained for this study. Each author certified that all investigations were conducted according to ethical principles. Written informed consent was obtained from all patients included in the study. We confirm that all methods have been performed according to the relevant guidelines and regulations.

## Therapeutic methods

### 1Preoperative therapy

All patients diagnosed with thoracic spinal TB were treated with HREZ (300 mg/d isoniazid, 450 mg/d rifampicin, 750 mg/d ethambutol and 1.5 g/d pyrazinamide) for at least 2 to 4 weeks before the operations.

### Operative technique

**Figure 1:**
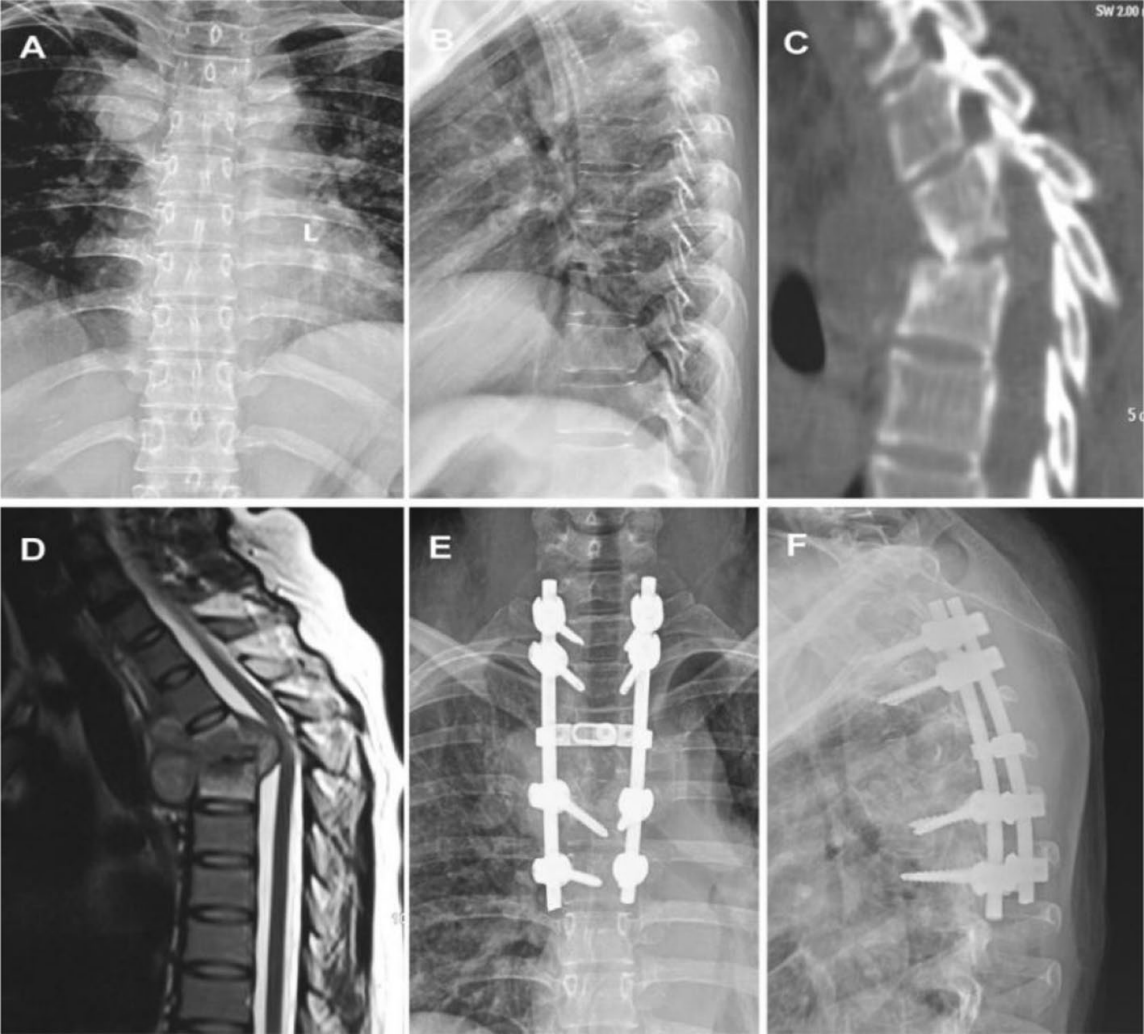
The patient (female; 43 years old) presented with thoracic tuberculosis at T3-4 and underwent single posterior debridement, bone graft fusion and instrumentation. (**A**, **B**): X-ray in the positive and lateral position before surgery; (**C**, **D**): preoperative CT and MRI examination; (**E**, **F**): x-ray in the positive and lateral position after surgery.

All patients were intubated under general anaesthesia. Patients were placed in a prone position and a posteromedial incision was made to expose the vertebral plate, facet joints and costovertebral joints. After locating the lesion using C-arm fluoroscopy, pedicle screws were inserted in the healthy vertebral body adjacent to the upper and lower affected vertebrae, followed by kyphosis correction. From the costovertebral joints or pedicles of the vertebral arch, the lesion in the anterior vertebral body was removed, as were as the dead bone, necrotic intervertebral disc and caseous necrotic tissue. An autogenous iliac bone graft was then embedded into the focal zone (Fig. 1). After the operation, conventional bacterial cultivation and pathological diagnosis were performed.

### Postoperative treatment

Prophylactic antibiotics were used for 72 h and oral administration of HREZ continued after the operation. Six months later, pyrazinamide was withdrawn while isoniazid, rifampicin and ethambutol (HRE) were administered daily for another 10 to 12 months. The drainage tube was removed when drainage volume was less than 50 ml/24 h. Afterward, the patients were permitted to walk with the support of a mobility aid. Initially, only non-weight-bearing activity was recommended. Normal weight-bearing activity was not allowed until the confirmation of intervertebral fusion with x-ray and CT examinations.

### Evaluation standard

Visual analogue scale (VAS) scores were used for pain assessment. The Frankel grading system was employed in the assessment of preoperative and postoperative spinal cord injuries. Erythrocyte sedimentation rate and CRP level were assessed to monitor disease activity. The preoperative and postoperative kyphosis angles were measured using the method proposed in a previous study^12^. X-ray and CT examinations were conducted to assess bone graft fusion, loss of correction angle and internal fixation failure^13^.

### Statistical analysis

The software SPSS 19.0 (SPSS, Inc., Chicago, IL) was used for data analysis. Wilcoxon’s signed-rank test was used to assess the differences between preoperative and postoperative American Spinal Injury Association (ASIA) impairment scale scores. The paired t-test was performed to compare the preoperative and postoperative degrees of kyphosis deformity and ESR and CRP levels. The Wilcoxon rank-sum test was employed in the analysis of discrepancy in normal distributions. *P* value < 0.05 was considered indicative of a significant difference.

## Results

Definite diagnosis was all established through bacterial cultivation and/or pathological diagnosis in the 88 patients and mycobacterium tuberculosis was found in 66. Of the 88 patients, 50 were male and 38 were female. The mean age was 44.6 ± 7.8 years (range, 22–69). The mean operation time was 165 ± 38 min. The mean intraoperative blood loss was 468 ± 103 mL. The mean length of hospital stay was 21 ± 4 days. The mean time of bone graft fusion was 6.6 ± 0.3 min. Varying degrees of paraspinal abscess around the muscle were seen in 14 patients. It took 7.2 ± 0.6 min (range, 6–12 m) for the abscess to disappear after the operation.

Follow ups were performed in all patients, with a mean duration of follow up in the study population of 40.6 ± 4.1 min (range, 36–48 m). Dehiscence of the wound in 1 patient healed 23 days after debridement and suturing. Another patient experienced postoperative sinus track formation and the sinus tract was treated weekly with vacuum sealing drainage and closed 4 weeks after the operation. In addition, Breakage of the internal fixation was found in 1 patient during a follow up 8 months after the operation. During the follow-up period, no recurrence of TB was reported.

Table 1 shows the preoperative and postoperative kyphosis angles, angle correction, angle loss and angle correction rates. The preoperative kyphosis angle was 26.5 ± 4.5. The postoperative and final follow-up kyphosis angles were both significantly smaller than the preoperative kyphosis angles (*P* < 0.05). The postoperative angle correction rate reached 81.5% and its postoperative angle loss was only 4.1%.

**Table 1:** Kyphosis correction and kyphosis lost in single posterior approach.

Preoperative kyphosis angle (°)*	Postoperation			Final follow-up		
	Kyphosis angle (°)	Angle correction (°)	Correction rate (%)	Kyphosis angle (°)	Angle lost (°)	Lost rate (%)
26.5 ± 4.5	5.6 ± 1.9	21.8 ± 5.4	81.5 ± 11.4	8.2 ± 2.7	1.1 ± 1.0	4.1 ± 4.8

All patients had neurological impairment before surgery (Frankel Grade C or D, Table 2). At the most recent follow up, 82 patients had neurofunctional parameters within the normal range. However, significant differences were found between the neurofunctional parameters before the operations and at the final follow up (*P* < 0.05).

**Table 2:** Neurological recovery according to Frankel grade.

Time point	A	B	C	D	E
Preoperative			64	24	
Final follow-up*				6	82

*P < 0.05 versus preoperative.

Table 3 summarizes the changes in VAS scores and in ESR and CRP levels 6 weeks after surgery and at the most recent follow up. The VAS scores and ESR and CRP levels 6 weeks after surgery and at the final follow up were significantly lower than before surgery (*P* < 0.05). In addition, the VAS scores and ESR and CRP levels were significantly lower than those 6 weeks after surgery (*P* < 0.05).

**Table 3:** Measures of surgical outcomes of the single posterior approach.

Measure	VAS			CRP (mg/L)			ESR (mm/h)		
	Pre-op	6 weeks post-op	Final follow up	Pre-op	6 weeks post-op	Final follow-up	Pre-op	6 weeks post-op	Final follow-up
	6.4 ± 0.6	2.2 ± 0.6*	0.6 ± 0.2^Δ^	20.4 ± 3.0	9.6 ± 1.1*	3.0 ± 0.3^Δ^	38.6 ± 69	21.0 ± 1.6*	9.5 ± 0.6^Δ^

VAS, visual analogue scale; ESR, erythrocyte sedimentation rate; CRP, C-reactive protein; Pre-op, Preoperative; Post-op, Postoperative.

*P < 0.05 versus preoperative.

^Δ^*P* < 0.05 versus 6 weeks’ postoperative.

## Discussion

The existing treatment for spinal TB includes primarily anti-TB drugs and surgical intervention. Anti-TB drug therapy plays an essential role in treating spinal TB and provides a basis for surgical treatment. The goals of surgical treatment are to eradicate the lesion(s), relieve spinal cord compression, correct kyphosis deformity and restabilize the spine^8^. Surgery alone, without regular anti-TB treatment, is extremely dangerous and ineffective. Effective surgical treatment is only possible in combination with effective anti-TB drug therapy. It is difficult to relieve spinal cord compression, improve nerve dysfunction and prevent progressive spinal deformity using conservative treatment alone. In contrast, surgery is an effective solution to this problem^14^.

The choice of surgical approach when accessing the lesion sites remains controversial. The single anterior approach has been widely accepted as the preferred treatment strategy for spinal TB treatment^15^, because it allows for thorough removal of lesions, decompression of the anterior structure of the spine, easy bone grafting, kyphosis correction and reconstruction of the spine under direct vision, with the lesions being fully exposed^16^. However, it shows inadequate fixation rigidity and orthopaedic strength^8, 9^ and imposes a high risk of vascular injury because of poor access to the lesion site^17^. Considering the huge exposure, the long wound and a major risk of hemopneumothorax, this method is not a good choice for patients with multisegmental thoracic spinal TB^18^. Other studies have reported that the anterior approach was associated with postoperative kyphosis angle loss^17, 18^.

As the surgical techniques in spinal TB develop, single posterior debridement, bone grafting and instrumentation have achieved satisfactory outcomes of kyphosis correction and reconstruction of the spine, as well as long-segment fixation without creating a significant wound^10, 11^. Zhang et al.^19^ also reported favourable clinical outcomes of thoracic spinal TB treatment using the single posterior approach. Liu et al.^20^ performed posterior debridement, bone grafting and instrumentation in patients with monosegmental thoracic spinal TB, which achieved a satisfactory curative effect, with favourable bone graft fusion and significant improvement in the patients’ Cobb angles. Hassan et al.^21^ treated thoracic spinal TB patients with the anterior or posterior approach and found that the posterior approach outperformed the anterior approach in mean operation time, intraoperative blood loss and blood transfusion. In addition, the single posterior approach produced favourable surgical outcomes by only creating a small wound to access the lesion site when treating thoracic spinal TB. In this study, the single posterior approach was performed in 88 patients, and they experienced significant improvement in ASIA impairment scale scores and a sharp decrease in Cobb angles and ESR levels after the operation. The preoperative and postoperative parameters were significantly different (*P* < 0.05), indicating favourable surgical outcomes with the posterior approach. However, the single posterior approach also has its own limitations. Specifically, it is difficult to remove TB lesions radically using the posterior approach. TB bacteria may invade the healthy tissue in the posterior structure of the spine and the surgery may damage the healthy posterior spine, compromise the spinal stability, interfere with the spinal cord and increase postoperative intraspinal scar adhesion. Beyond that, this approach only gives limited exposure of the anterior structure of the spine and thus, it is not an option when there is a large paraspinal abscess. As reported in our previous study, the single posterior approach had a shorter operation time and a smaller volume of intraoperative blood loss, compared with the anterior approach^8^ and those findings are proven in this study. Through analysis, it is thought that these advantages of the posterior approach are associated with the development of the pedicle screw insertion technique. In addition, the posterior approach itself is widely used in many short-segment fixation procedures (no more than 3 segments). Furthermore, the posterior approach had an angle loss rate lower than that of the anterior approach in terms of kyphosis correction, which was reported in another relevant study^19^. The rationale for using the posterior approach lies in the removal of the lesion and the sclerotic bone surrounding the lesion to clear the path for anti-TB drugs. the residual TB-like lesion and purulent fluid require long-term, standard anti-TB chemotherapy to achieve an effective postoperative solution. A prospective multicentre randomized comparative study with a larger sample size is needed to investigate the long-term efficacy of the single posterior approach.

In conclusion, single posterior debridement, bone grafting and instrumentation combined with effective standard anti-TB chemotherapy produce satisfactory clinical outcomes in the treatment of thoracic spinal TB. Moreover, the single posterior approach has many advantages, including shorter operation time and less intraoperative blood loss, compared with the single anterior approach. In addition, it clearly outperforms the single anterior approach in kyphosis correction, angle correction rate and angle loss. All bone grafts showed fusion. To date, the patients are well according to clinical and imaging follow-up results. Despite the preliminary results from the medium-term follow up, it is necessary to perform a study based on long-term follow up.
